# Clinical characteristics and influencing factors of adverse outcomes following clinical pregnancy after laparoscopic tubal anastomosis

**DOI:** 10.1080/07853890.2025.2564283

**Published:** 2025-10-06

**Authors:** Yan Guo, Xiaochuan Liu, Yuanyuan Zhang, Yunmei Ke, Jia Liu, Jiahong Tan, Yun Zhu, Na Ma, Yonghan Luo, Jie Zhang

**Affiliations:** a Department of Reproductive Gynecology, NHC Key Laboratory of Healthy Birth and Birth Defect Prevention in Western China, First People’s Hospital of Yunnan Province, Kunming, Yunnan, China; b Department of Reproductive Gynecology, The Affiliated Hospital of Kunming University of Science and Technology, Kunming, Yunnan, China; c Faculty of Life Science and Technology, Kunming University of Science and Technology, Kunming, Yunnan, China; d Clinical Research Center, Shanghai Baoshan Luodian Hospital, Shanghai, China; e The First Clinical Medical School, Kunming Medical University, Kunming, Yunnan, China; f Second Department of Infectious Disease, Kunming Children’s Hospital, Kunming, Yunnan, China; g Yunnan Key Specialty of Pediatric Infection (Training and Education Program)/Kunming Key Specialty of Pediatric Infection, Kunming, Yunnan, China

**Keywords:** Laparoscopic tubal anastomosis, pregnancy loss, age, BMI, reproductive outcomes

## Abstract

**Objective:**

To identify clinical characteristics and risk factors for adverse pregnancy outcomes after clinical pregnancy following laparoscopic tubal anastomosis (LTA).

**Methods:**

Retrospective analysis of 253 patients undergoing LTA (May 2016–December 2022) achieving clinical pregnancy. Patients were categorized into pregnancy loss (*n* = 70) and live birth groups (*n* = 183). Univariate/multivariate logistic regression identified risk factors. Restricted cubic splines (RCS) explored dose-response relationships. Receiver operating characteristic (ROC) and decision curve analysis (DCA) assessed prediction.

**Results:**

The majority of patients achieved successful delivery, with a live birth rate of 72.3%. However, the overall pregnancy loss rate was 27.7%, including an ectopic pregnancy incidence of 10.7% (27/253) and a miscarriage rate of 17.0% (43/253). Advanced maternal age and elevated body mass index (BMI) were identified as independent risk factors for pregnancy loss after LTA (age: OR = 1.123, 95% CI: 1.010–1.253; BMI: OR = 1.136, 95% CI: 1.030–1.256). ROC analysis demonstrated that both age and BMI exhibited moderate discriminative ability for pregnancy loss (AUC: age = 0.723, BMI = 0.724). RCS analysis indicated a linear relationship between age and pregnancy loss (*p* = 0.464) but a nonlinear association for BMI (*p* < 0.001). DCA confirmed the clinical utility of age and BMI in predicting pregnancy loss within a specific high-risk threshold range.

**Conclusion:**

Advanced maternal age and higher BMI significantly increase the risk of pregnancy loss after LTA. These findings highlight the need for individualized preoperative counseling and postoperative monitoring to optimize reproductive outcomes in this patient population.

## 1. Introduction

Tubal ligation (TL) is a widely used permanent contraceptive method, with a failure rate of less than 0.5% and being the preferred choice for approximately 18% of contraceptive users [1]. However, with changes in reproductive policies and personal family planning, 20–30% of women seek to conceive again after sterilization [2–4]. In such cases, tubal anastomosis (TA) and *in vitro* fertilization with embryo transfer (IVF-ET) are two primary options for achieving pregnancy [5,6]. However, as TA is a more cost-effective and physiologically favorable option, the American Society for Reproductive Medicine (ASRM) has consistently recommended microsurgical TA as the preferred technique for tubal ligation reversal [7–9].

The first TA procedure was performed *via* laparotomy in 1967 [10]. Since then, surgical advancements have introduced laparoscopic and robotic techniques, with laparoscopic tubal anastomosis (LTA) emerging as a widely practiced method for reversing TL [11]. Reported pregnancy rates following LTA range from 55.2 to 75.3% [12]. However, despite the significant clinical success of LTA, there remains considerable heterogeneity in postoperative pregnancy outcomes. Some patients experience adverse pregnancy outcomes, such as miscarriage and ectopic pregnancy, following clinical pregnancy, which poses substantial challenges to both fertility prognosis and mental health [12]. Therefore, a deeper understanding of the factors influencing adverse pregnancy outcomes after LTA has significant clinical implications.

Currently, research on pregnancy outcomes following LTA, both domestically and internationally, has primarily focused on surgical success rates and pregnancy rates, with limited analysis of clinical characteristics and risk factors associated with adverse pregnancy outcomes [13]. With the increasing trend of advanced maternal age, there is a lack of systematic studies on whether factors such as age and obesity influence pregnancy outcomes after LTA. Existing literature suggests that advanced maternal age and elevated body mass index (BMI) may increase the risk of pregnancy loss in both natural conception and assisted reproductive technologies (ART) [14–16]. However, the role of these factors in pregnancy outcomes following LTA remains to be further investigated.

Therefore, identifying risk factors for pregnancy loss after LTA is of great clinical significance, as it not only aids in precise preoperative patient selection and individualized counseling but also directly informs postoperative pregnancy management strategies. Addressing this critical clinical need, our study retrospectively analyzed data from 640 patients who underwent LTA at The First People’s Hospital of Yunnan Province between May 2016 and December 2022, ultimately including 253 patients with confirmed clinical pregnancies. Using advanced statistical methods, including multivariate logistic regression and restricted cubic spline (RCS) models, we systematically explored the impact of key factors such as age and BMI on pregnancy outcomes. Notably, our findings highlight the need for enhanced preoperative assessment and intensified postoperative monitoring for older patients, while targeted weight management interventions may improve pregnancy prognosis for obese patients. Furthermore, by uncovering potential dose-response relationships between age, BMI, and pregnancy loss, this study provides a scientific basis for developing a more precise clinical risk stratification system. These findings not only fill a critical gap in research on factors influencing pregnancy outcomes after LTA but also offer valuable evidence-based insights to optimize patient management and improve reproductive prognosis.

## 2. Materials and methods

### 2.1. Study population

**Figure 1.**
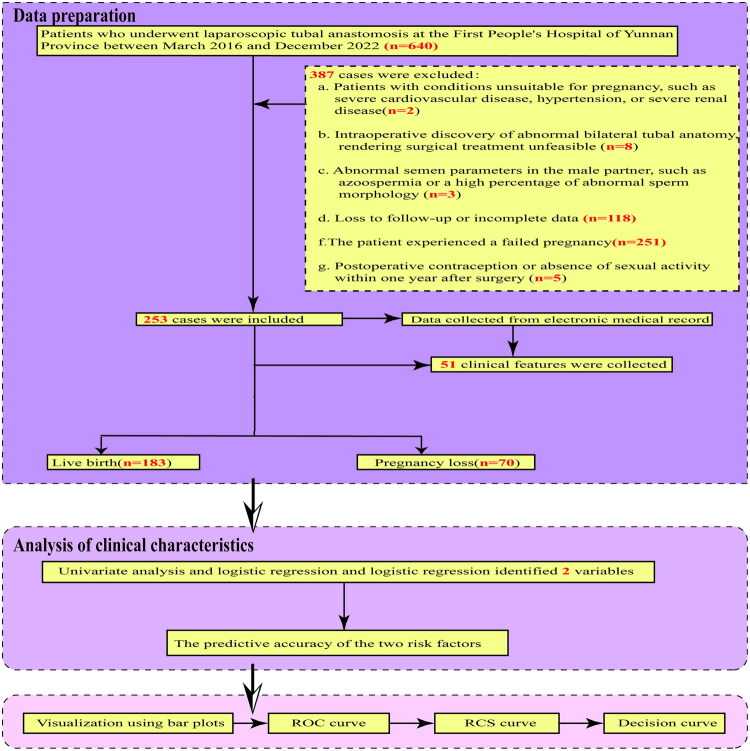
Study flowchart of clinical characteristics and influencing factors of adverse outcomes following clinical pregnancy after laparoscopic tubal anastomosis. Abbreviation: receiver operating characteristic (ROC) curve; restricted cubic spline (RCS).

A retrospective review was performed on the clinical records of 640 patients who underwent LTA at the First People’s Hospital of Yunnan Province from May 2016 to December 2022 (Study flowchart presented in Figure 1). Ethical approval for this study was granted by the Ethics Committee of the First People’s Hospital of Yunnan Province (Approval No. KHLL2023-KY120). Due to the retrospective design of the study, the requirement for informed consent was waived by the ethics committee of Ethics Review Committee of the First People’s Hospital of Yunnan Province. In addition, all methods were carried out in accordance with relevant guidelines and regulations or Declaration of Helsinki.

### 2.2. Exclusion criteria

Exclusion criteria:

- Patients with medical conditions contraindicating pregnancy, such as severe cardiovascular disease, hypertension, or advanced renal disease;
- Intraoperative discovery of significant bilateral tubal abnormalities rendering surgical intervention impractical;
- Male partner with abnormal semen parameters, including azoospermia or a high proportion of morphologically abnormal sperm;
- Patients lost to follow-up or with incomplete clinical data;
- Pregnancy failure (defined as the absence of pregnancy despite engaging in regular unprotected intercourse for two years following LTA surgery);
- Postoperative use of contraception or lack of sexual activity within one year following surgery.

All patients were followed up for two years after LTA. Those who achieved a successful live birth within this period were classified into the Live Birth Group, while those who experienced an ectopic pregnancy or miscarriage were categorized into the Pregnancy Loss Group.

### 2.3. Exclusion criteria data extraction

Clinical data were obtained from the hospital’s electronic medical records system and supplemented by telephone follow-ups to gather patients’ postoperative information.

### 2.4. Key definitions

Normal frequency of sexual activity: two to three times per week.

Duration of sterilization: the time interval between tubal ligation (TL) and tubal anastomosis (TA).

Pregnancy loss: the spontaneous or induced termination of pregnancy before the fetus reaches viability, typically before 24 weeks of gestation. This term encompasses miscarriage (spontaneous abortion), ectopic pregnancy, stillbirth, and other forms of fetal demise.

### 2.5. Statistical analysis

Statistical analysis was conducted using R software (version 4.4.1). For continuous variables following a normal distribution, *t*-tests were applied, with data presented as mean ± standard deviation (mean ± SD). Non-normally distributed data were analyzed using the Mann-Whitney *U* test and expressed as median and interquartile range [M (P25, P75)]. Categorical variables were assessed using the *χ*^2^ test or Fisher’s exact test, with results reported as counts (*n*) and percentages (%).

Data visualization was performed using the ‘ggplot’ function in R. Initially, univariate analysis was conducted to identify potential influencing factors of adverse outcomes following clinical pregnancy after LTA. Statistically significant risk factors (*p* < 0.05) from the univariate analysis were then incorporated into a multivariate logistic regression model. For variable selection, we followed a stepwise selection approach based on the Akaike Information Criterion (AIC) to ensure optimal model fit. In cases of missing data, multiple imputation was performed to handle missing values and avoid bias due to incomplete data. A bar chart was used to visualize the final risk factors.

Additionally, a restricted cubic spline (RCS) approach combined with logistic regression was employed to flexibly model the relationship between risk factors and outcomes. The risk factors were treated as continuous variables, with four knots (P5, P35, P65, and P95) selected based on their percentile distribution. The spline function was fitted using the “rms” package in R.

Receiver operating characteristic (ROC) curve analysis was performed to assess the predictive capability of these risk factors for pregnancy outcomes. Furthermore, decision curve analysis (DCA) was conducted to evaluate the clinical utility of these risk factors. A *P*-value < 0.05 was considered statistically significant.

## 3. Results

### 3.1. Clinical features of included patients

#### 3.1.1. General information

In this study, a total of 640 patients who underwent LTA surgery were initially enrolled. Following the application of exclusion criteria, 387 cases were excluded for the following reasons: a) Patients with conditions contraindicating pregnancy, such as severe cardiovascular disease, hypertension, or severe renal disease (*n* = 2); b) Intraoperative discovery of abnormal bilateral tubal anatomy, rendering surgical intervention unfeasible (*n* = 8); c) Abnormal semen parameters in the male partner, including azoospermia or a high percentage of abnormal sperm morphology (*n* = 3); d) Loss to follow-up or incomplete data (*n* = 118); e) Failure to achieve a pregnancy (*n* = 251); f) Postoperative use of contraception or lack of sexual activity within one year following surgery (*n* = 5). Consequently, 253 patients remained eligible for analysis, which were subsequently categorized into the Pregnancy Loss Group (*n* = 70) and the Live Birth Group (*n* = 183). (see Figure 1). The detailed clinical characteristics and intergroup differences are presented in Table 1.

**Table 1.** Comparison of clinical characteristics between the live birth group and the pregnancy loss group after laparoscopic tubal anastomosis.

	Total (*n* = 253)	Live birth group (*n* = 183)	Pregnancy loss group (*n* = 70)	*p*
General information				
Age (median (IQR)), y	35 (32, 38)	35 (32, 37)	36 (33, 38)	< 0.001
Ethnic groups, n (%)				0.773
Han ethnicity	200 (79)	146 (80)	54 (77)	
Others	53 (21)	37 (20)	16 (23)	
BMI (median (IQR)), kg/m²	22.37 (20.44, 24.14)	22.19 (20.2, 24.03)	23 (20.97, 25.5)	< 0.001
Height (median (IQR)), m	1.58 (1.55, 1.6)	1.56 (1.54, 1.6)	1.59 (1.55, 1.63)	0.007
Weight (median (IQR)), kg	55 (50, 61)	54 (50, 60)	58.5 (55, 63.75)	< 0.001
Profession, n (%)				0.568
Agricultural and labor workers	100 (40)	76 (42)	24 (34)	
Non-fixed occupation	77 (30)	52 (28)	25 (36)	
Fixed occupation	18 (7)	12 (7)	6 (9)	
No occupation or unspecified	58 (23)	43 (23)	15 (21)	
Time from surgery to clinical pregnancy (median (IQR)), m	6 (4, 13)	7 (4, 14)	6 (3, 12)	0.605
Reproductive history				
Number of full-term birth, (median(IQR)), *n*	2.00 (2.00, 2.00)	2.00 (2.00, 2.00)	2.00 (2.00, 2.00)	0.344
Number of preterm birth, (median(IQR)), *n*	0.00 (0.00, 0.00)	0.00 (0.00, 0.00)	0.00 (0.00, 0.00)	0.041
Number of miscarriage, (median(IQR)), *n*	0.00 (0.00, 1.00)	0.00 (0.00, 1.00)	1.00 (0.00, 2.00)	0.011
Number of living children, (median(IQR)), *n*	2.00 (2.00, 2.00)	2.00 (2.00, 2.00)	2.00 (2.00, 2.00)	0.723
Ectopic pregnancy, *n* (%)				1
NO	250 (99)	181 (99)	69 (99)	
YES	3 (1)	2 (1)	1 (1)	
Endocrine disease history				
Polycystic ovary syndrome, *n* (%)				0.292
NO	243 (96)	174 (95)	69 (99)	
YES	10 (4)	9 (5)	1 (1)	
Thyroid dysfunction, *n* (%)				0.732
NO	242 (96)	174 (95)	68 (97)	
YES	11 (4)	9 (5)	2 (3)	
Laboratory examination				
E2, (median (IQR)), pmol/L	279 (187, 508)	280.47 (186.25, 517)	257 (192.5, 464.5)	0.962
LH, (median (IQR)), IU/L	4.18 (3.24, 6.2)	4.06 (3.19, 5.98)	4.36 (3.52, 6.64)	0.25
FSH, (median (IQR)), IU/L	4.86 (4.01, 6.09)	4.78 (3.93, 5.94)	5.07 (4.3, 6.71)	0.126
PRL, (median (IQR)), mIU/L	199.74 (148.87, 308.73)	202.25 (147.47, 331.81)	195.74 (149.47, 259.82)	0.493
Prog, (median (IQR)), nmol/L	0.30(0.30, 0.30)	0.30(0.30, 0.30)	0.30(0.30, 0.30)	0.453
Testo, (median (IQR)), nmol/L	1.08 ± 0.32	1.09 ± 0.3	1.07 ± 0.36	0.79
TSH, (median (IQR)), mIU/L	1.95 (1.4, 2.77)	1.94 (1.4, 2.71)	1.97 (1.39, 3)	0.622
T3, (median (IQR)), nmol/L	100.72 ± 16.97	102.04 ± 17.38	96.74 ± 15.18	0.048
T4, (median (IQR)), nmol/L	1.72 ± 0.27	1.73 ± 0.27	1.69 ± 0.28	0.309
HBsAg, *n* (%)				0.47
NO	243 (96)	177 (97)	66 (94)	
YES	10 (4)	6 (3)	4 (6)	
HBsAb, *n* (%)				0.368
NO	183 (72)	129 (70)	54 (77)	
YES	70 (28)	54 (30)	16 (23)	
HBeAg, *n* (%)				0.307
NO	249 (98)	181 (99)	68 (97)	
YES	4 (2)	2 (1)	2 (3)	
HBeAb, *n* (%)				0.946
NO	229 (91)	165 (90)	64 (91)	
YES	24 (9)	18 (10)	6 (9)	
HBcAb, *n* (%)				0.692
NO	222 (88)	162 (89)	60 (86)	
YES	31 (12)	21 (11)	10 (14)	
HCV-Ab, *n* (%)				1
NO	253 (100)	183 (100)	70 (100)	
HIV-Ab, *n* (%)				1
NO	252 (100)	182 (100)	70 (100)	
TPPA, *n* (%)				0.326
NO	248 (98)	178 (97)	70 (100)	
YES	5 (2)	5 (3)	0 (0)	
Surgical indications				
Duration of sterilization (median (IQR)), y	10 (7, 12)	10 (7, 12)	10 (8, 13)	0.035
Reason for surgery, *n* (%)				0.814
Desire for more children	107 (42)	79 (43)	28 (40)	
Remarriage	11 (4)	8 (4)	3 (4)	
Child’s death	133 (53)	95 (52)	38 (54)	
Others	2 (1)	1 (1)	1 (1)	
Surgical Characteristics				
Surgical physician’s working years (median (IQR)), y	16 (12, 25)	16 (12, 24.5)	17 (10.5, 25)	0.739
The duration of the surgical intervention (median (IQR)), min	90 (75, 120)	90 (75, 120)	90 (75, 120)	0.792
Surgical approach, *n* (%)				1
Laparoscopy	253 (100)	183 (100)	70 (100)	
Ligation site				
Left fallopian tube, *n* (%)				0.601
Interstitial part	4 (2)	2 (1)	2 (3)	
Interstitial-Isthmic part	2 (1)	1 (1)	1 (1)	
Isthmic part	107 (42)	80 (44)	27 (39)	
Isthmic-Ampullary part	47 (19)	34 (19)	13 (19)	
Ampullary part	91 (36)	65 (36)	26 (37)	
Unclear	2 (1)	1 (1)	1 (1)	
Right fallopian tube, *n* (%)				0.359
Interstitial part	4 (2)	3 (2)	1 (1)	
Interstitial-Isthmic part	2 (1)	1 (1)	1 (1)	
Isthmic part	102 (40)	77 (42)	25 (36)	
Isthmic-Ampullary part	49 (19)	32 (17)	17 (24)	
Ampullary part	95 (38)	70 (38)	25 (36)	
Unclear	1 (0)	0 (0)	1 (1)	
Postoperative patency status				
Left fallopian tube, *n* (%)				0.516
Patent	231 (92)	1667(91)	66 (94)	
Partially patent	18 (7)	15 (8)	3 (4)	
Complete obstruction	2 (1)	1 (1)	1 (1)	
Right fallopian tube, *n* (%)				0.933
Patent	224 (88)	162 (88)	62 (89)	
Partially patent	27 (11)	19 (10)	8 (11)	
Complete obstruction	2 (1)	2 (1)	0 (0)	
Comorbidities				
Uterine fibroids, *n* (%)				0.281
NO	235 (93)	172 (94)	63 (90)	
YES	18 (7)	11 (6)	7 (10)	
Adenomyosis, *n* (%)				0.171
NO	232 (92)	171 (93)	61 (87)	
YES	21 (8)	12 (7)	9 (13)	
Uterine malformations, *n* (%)				0.186
NO	250 (99)	182 (99)	68 (97)	
YES	3 (1)	1 (1)	2 (3)	
Uterine adhesions, *n* (%)				0.578
NO	249 (98)	179 (98)	70 (100)	
YES	4 (2)	4 (2)	0 (0)	
Endometrial polyps, *n* (%)				0.339
NO	223 (88)	164 (90)	59 (84)	
YES	30 (12)	19 (10)	11 (16)	
Hydrosalpinx, *n* (%)				1
NO	242 (96)	175 (96)	67 (96)	
YES	11 (4)	8 (4)	3 (4)	
Mesosalpinx cyst, *n* (%)				1
NO	149 (59)	108 (59)	41 (59)	
YES	104 (41)	75 (41)	29 (41)	
Pelvic adhesions, *n* (%)				1
NO	33 (13)	24 (13)	9 (13)	
YES	220 (87)	159 (87)	61 (87)	
Ovarian cysts, *n* (%)				0.732
NO	242 (96)	174 (95)	68 (97)	
YES	11 (4)	9 (5)	2 (3)	
Endometriosis, *n* (%)				1
NO	251 (99)	181 (99)	70 (100)	
YES	2 (1)	2 (1)	0 (0)	
Postoperative sexual life survey				
Time to resume sexual activity after surgery (median (IQR)), m	3.00(2.00, 4.00)	3.00(2.00, 4.00)	3.00(2.00, 4.00)	0.73
Frequency of sexual activity, *n* (%)				0.277
Normal	1 (0)	0 (0)	1 (1)	
Abnormal	252 (100)	183 (100)	69 (99)	

Abbreviation: IQR, interquartile range; n, number; BMI, body mass index; E2, estradiol; LH, luteinizing hormone; FSH, follicle-stimulating hormone; PRL, prolactin; Prog, progesterone; Testo, testosterone; TSH, thyroid-stimulating hormone; T3, triiodothyronine; T4, thyroxine; HBsAg, hepatitis B surface antigen; HBsAb, hepatitis B surface antibody; HBeAg, hepatitis B e antigen; HBeAb, hepatitis B e antibody; HBcAb, hepatitis B core antibody; HCV-Ab, hepatitis C antibody; HIV-Ab, HIV antibody; TPPA, treponema pallidum particle Agglutination; ART, Assisted reproductive technology.

Among the 253 patients who achieved clinical pregnancy, the median time from surgery to clinical pregnancy was six months, with no statistically significant difference between the groups. The median age was 35 years (IQR: 32–38), and patients in the Pregnancy Loss Group were significantly older than those in the Live Birth Group (*p* < 0.001). Additionally, BMI was notably higher in the Pregnancy Loss Group (*p* < 0.001). No statistically significant differences were observed in occupational distribution between the groups.

**Figure 2.**
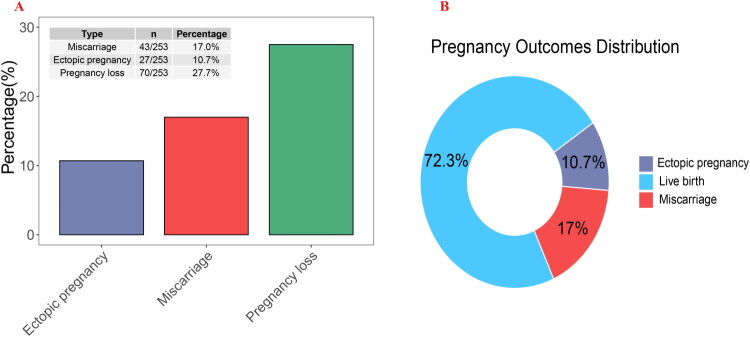
Pregnancy outcomes following laparoscopic tubal anastomosis. (**A**) Bar chart depicting the percentage of adverse pregnancy outcomes. (**B**) Donut chart illustrating the distribution of pregnancy outcomes.

Regarding pregnancy outcomes, the majority of patients achieved successful delivery, with a live birth rate of 72.3%. However, the overall pregnancy loss rate was 27.7%, including an ectopic pregnancy incidence of 10.7% (27/253) and a miscarriage rate of 17.0% (43/253) (Figure 2).

#### 3.1.2. Reproductive history

The number of full-term births showed no statistically significant difference between the two groups (*p* = 0.344), the incidence of preterm birth (*p* = 0.041) and the number of miscarriages (*p* = 0.011) were significantly higher in the Pregnancy Loss Group. Additionally, there was no significant difference in the number of live births between the two groups (*p* = 0.723).

#### 3.1.3. Endocrine disease history

The distribution of polycystic ovary syndrome (PCOS) showed no significant difference between the two groups (*p* = 0.292), and the incidence of thyroid dysfunction was also not statistically different (*p* = 0.732).

#### 3.1.4. Laboratory examination

There were no significant differences in hormone levels, including estradiol (E2), luteinizing hormone (LH), follicle-stimulating hormone (FSH), prolactin (PRL), progesterone (Prog), testosterone (Testo), and thyroid-stimulating hormone (TSH) (*p* > 0.05). However, triiodothyronine (T3) levels were significantly lower in the adverse outcome group (*p* = 0.048).

#### 3.1.5. Surgical-related factors

The duration of sterilization was longer in the pregnancy loss group (*p* = 0.035). However, the surgeon’s years of experience (*p* = 0.739) and the duration of surgical intervention (*p* = 0.792) did not show significant effects. Furthermore, all patients underwent surgery exclusively *via* laparoscopy.

#### 3.1.6. Tubal anatomy and patency status

Postoperative tubal patency rates were relatively high, with the left fallopian tube achieving a patency rate of 92% (231/253) and the right fallopian tube 88% (224/253). There was no significant difference in patency status between the two groups (left: *p* = 0.516, right: *p* = 0.933). Additionally, the location of tubal ligation showed no significant difference between the groups, suggesting that the anatomical site may not be a major factor affecting pregnancy outcomes.

#### 3.1.7. Comorbidities

There were no significant differences in the incidence of uterine fibroids (*p* = 0.281), adenomyosis (*p* = 0.171), uterine malformations (*p* = 0.186), intrauterine adhesions (*p* = 0.578), endometrial polyps (*p* = 0.339), or pelvic adhesions (*p* = 1) between the two groups.

### 3.2. Diagnostic value of age and BMI for pregnancy loss after LTA

**Figure 3.**
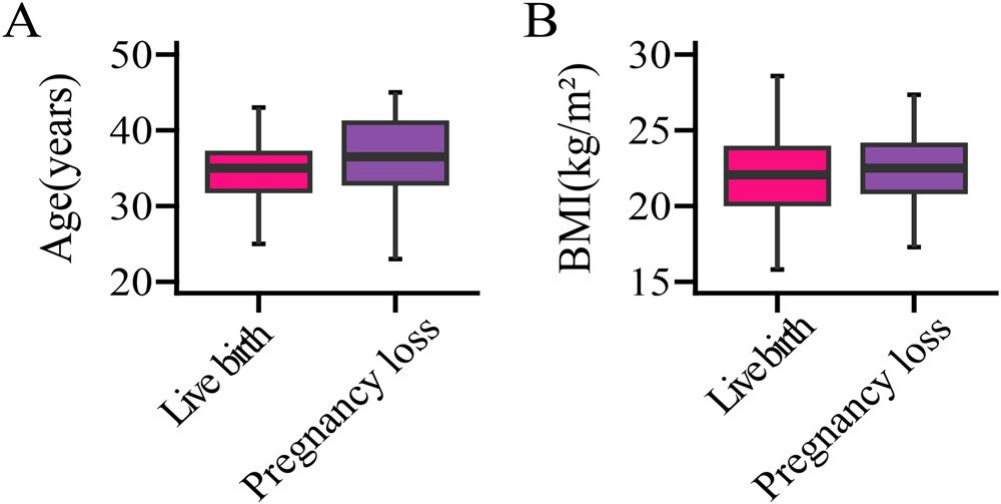
Comparison of maternal age and BMI between pregnancy loss and live birth groups following laparoscopic tubal anastomosis.

**Figure 4.**
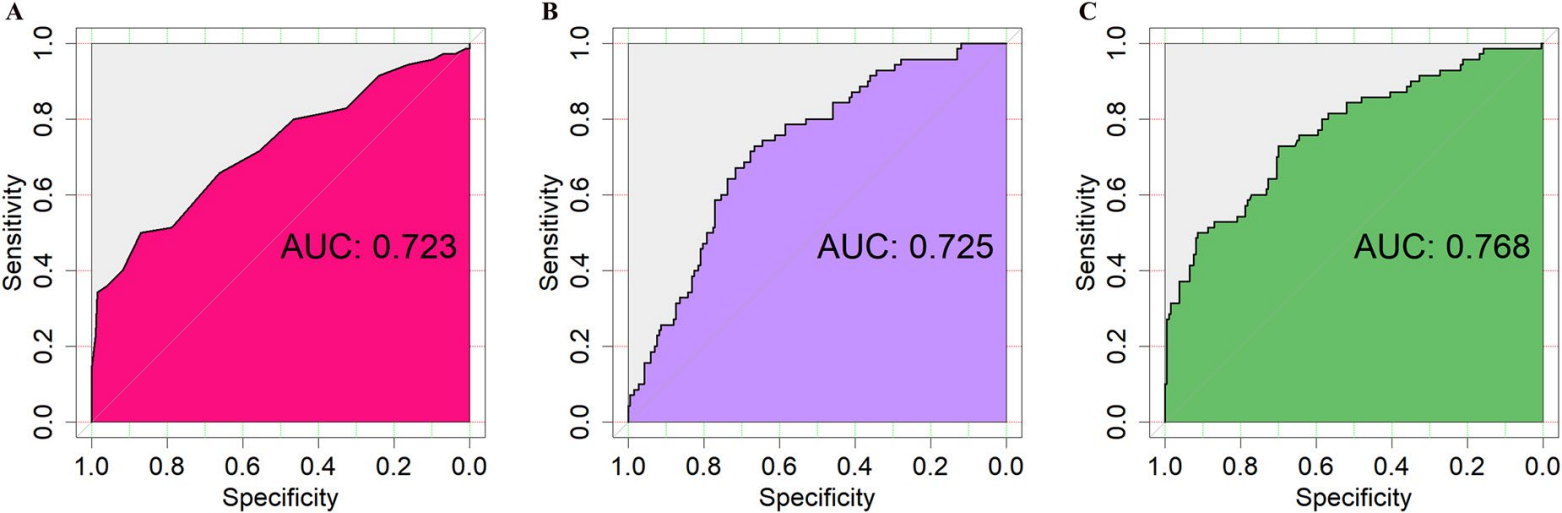
Receiver operating characteristic (ROC) curves for predicting adverse pregnancy outcomes following laparoscopic tubal anastomosis. (**A**) maternal age, (**B**) BMI.

Univariate analysis identified six variables with statistically significant differences between the two groups: age, BMI, number of preterm births, number of miscarriages, T3 levels, and duration of sterilization (see Table 1). Multivariate logistic regression analysis of these six variables revealed that only age and BMI were independent prognostic factors for pregnancy loss after LTA, with an OR of 1.123 (95% CI: 1.010–1.253) and 1.136 (95% CI: 1.030–1.256), respectively. Figure 3 presented a bar chart illustrating the differences in age and BMI between the pregnancy loss group and the live birth group. ROC curves were used to assess the predictive accuracy of age and BMI for pregnancy loss after LTA. Figure 4 demonstrated that the area under the ROC curve (AUC) for age was 0.723 (95% CI: 0.647–0.799) (Figure 4A) and for BMI was 0.724 (95% CI: 0.657–0.792) (Figure 4B), indicating a moderate predictive accuracy However, both age and BMI, as individual predictors of pregnancy loss after LTA, showed low predictive value. We further combined age and BMI to predict pregnancy loss after LTA, resulting in an AUC of 0.768 (95% CI: 0.700–0.836), which significantly improved the predictive accuracy (Figure 4C).

### 3.3. Dose-response analysis of age and BMI for pregnancy loss after LTA

**Figure 5.**
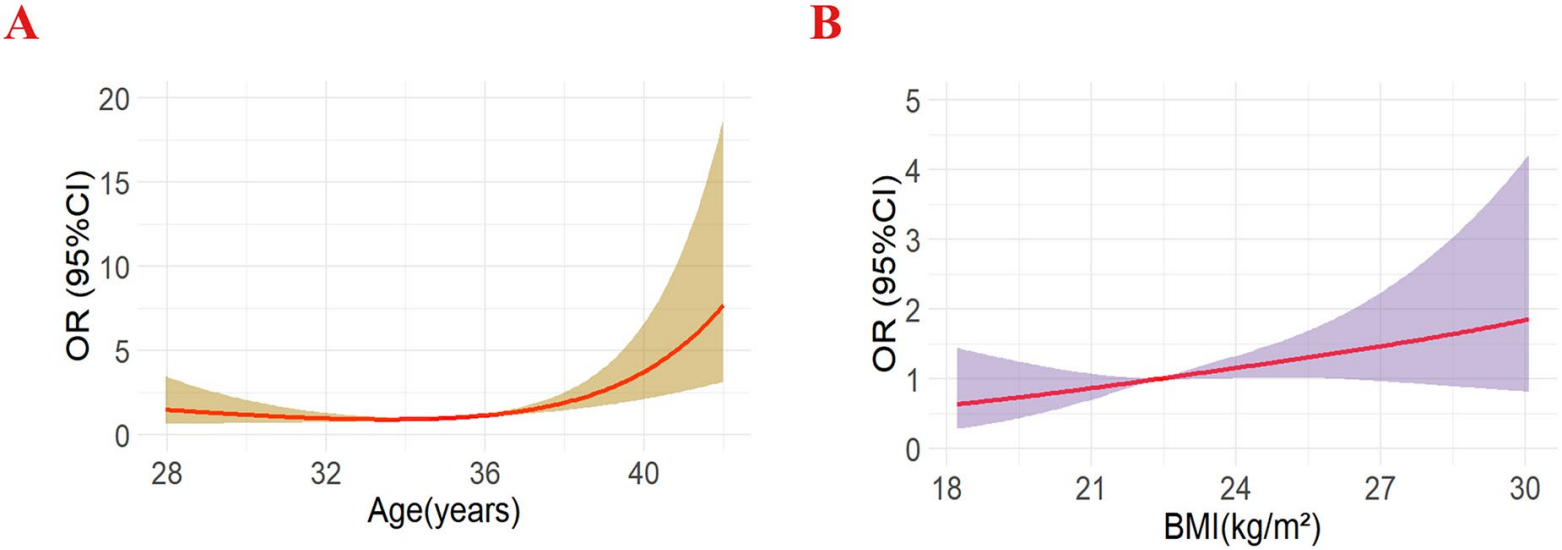
Association of age and BMI with adverse outcome following clinical pregnancy after laparoscopic tubal anastomosis. (**A**) Restricted Cubic Spline showing the odds ratio (OR) and 95% confidence interval (CI) for adverse pregnancy outcomes in relation to maternal age. The red line represents the OR, and the shaded area indicates the 95% CI. A sharp increase in OR is observed after the age of 35 years. (**B**) Spline plot depicting the relationship between BMI and adverse pregnancy outcomes. The red line indicates the OR, and the shaded area shows the 95% CI. The OR increases progressively with BMI, with a steeper rise observed beyond a BMI of approximately 25 kg/m^2^.

The dose-response relationship between age and BMI and pregnancy loss was analyzed using the restricted cubic spline method, integrating spline functions with logistic regression **(**Figure 5). A linear dose-response relationship was observed between age and pregnancy loss after LTA (nonlinearity test, *p* = 0.464). In contrast, the association between BMI and pregnancy loss after ALT was nonlinear (*p* < 0.001) (Figure 5A, B).

### 3.4. Decision curve analysis of age and BMI for pregnancy loss after LTA

**Figure 6.**
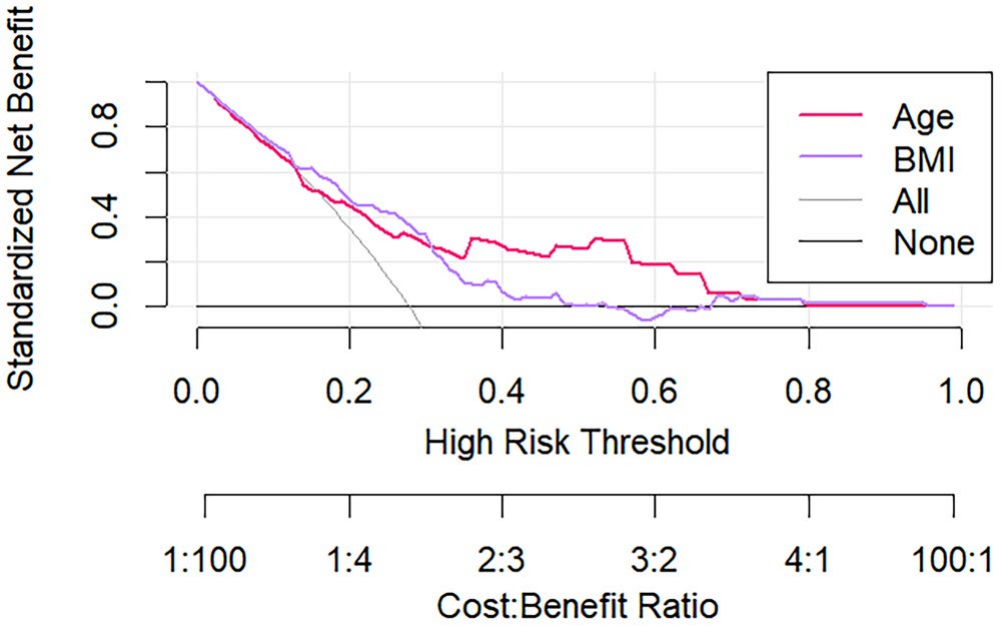
Decision curve analysis for predicting adverse pregnancy outcomes following laparoscopic tubal anastomosis. The red line represents the predictive model using maternal age, and the purple line represents the model using BMI.

The decision curve was constructed with net benefit on the *y*-axis and high-risk threshold on the *x*-axis, with the medium-high risk threshold set between 0 and 1. As shown in Figure 6, when the high-risk threshold ranged from 0.1 to 0.8 for age and 0.1–0.5 for BMI, the net benefit remained greater than zero. This indicateed that age and BMI possess significant clinical utility in predicting pregnancy loss after LTA.

## 4. Discussion

This retrospective study analyzed 253 patients who achieved clinical pregnancy following LTA, investigating post-operative pregnancy outcomes and associated risk factors. The findings revealed that although 72.3% of patients successfully conceived and delivered, 27.7% experienced pregnancy loss, including 10.7% ectopic pregnancies and 17.0% miscarriages. Multivariate analysis identified advanced maternal age and elevated BMI as independent risk factors for adverse pregnancy outcomes after LTA. ROC curve analysis demonstrated moderate predictive accuracy for both age (AUC = 0.723) and BMI (AUC = 0.724) in forecasting pregnancy loss. RCS modeling elucidated a linear dose-response relationship between advancing age and pregnancy loss (*p* = 0.464), while revealing a non-linear association for BMI (*p* < 0.001). DCA further validated the clinical utility of these predictive factors within specific high-risk threshold ranges, supporting their application in risk stratification and clinical decision-making.

The findings of this study indicate that patients undergoing LTA continue to face a notable risk of adverse pregnancy outcomes, primarily manifesting as ectopic pregnancy and spontaneous miscarriage. Although LTA, as a minimally invasive recanalization technique, effectively restores the anatomical continuity of the fallopian tubes and partially recovers reproductive capacity, clinical observations suggest that complications during subsequent pregnancy cannot be overlooked. Ectopic pregnancy is commonly attributed to postoperative alterations in tubal structure and function [17]. While LTA reestablishes tubal patency, fibrosis and scar formation at the anastomosis site often result in luminal narrowing or impaired peristalsis, thereby interfering with the normal transport of fertilized ova [18]. Furthermore, diminished ciliary activity and disruption of the tubal microenvironment are also key mechanisms leading to abnormal implantation within the fallopian tube [19]. In contrast, spontaneous miscarriage is more frequently associated with maternal factors. Advanced maternal age increases the likelihood of embryonic aneuploidy, compromising pregnancy maintenance, and declining embryo quality is another major contributor [20], In addition, postoperative reductions in endometrial receptivity, persistent local inflammation, and microenvironmental changes may interfere with embryo implantation and trigger miscarriage [21,22]. Previous literature reports that the incidence of ectopic pregnancy following LTA ranges from 1.7 to 14.3%, and the miscarriage rate ranges from 2.9 to 25%, which is consistent with the findings of this study [12]. In summary, while LTA plays a critical role in restoring the natural fertility potential of women of reproductive age, it still carries inherent postoperative risks, particularly for ectopic pregnancy and miscarriage. The pathophysiological mechanisms underlying adverse pregnancy outcomes following LTA remain incompletely understood, especially in terms of local tubal functional remodeling, endometrial receptivity alterations, and immune microenvironmental imbalance, all of which warrant further systematic investigation.

This study further found that advanced age and elevated BMI significantly increase the risk of pregnancy loss after LTA. Advanced age can lead to morphological and ultrastructural changes in the fallopian tubes, which may impair fertilization capacity [19]. As women grow older, there is a progressive decline in ovarian reserve, a reduction in oocyte quality, and a heightened risk of chromosomal abnormalities, all of which contribute to decreased pregnancy success rates [23]. Furthermore, research indicates that aging is linked to a slower basal metabolic rate, increased inflammatory responses, and disruptions in blood coagulation. These physiological changes may alter the endometrial environment, potentially impairing embryo implantation and pregnancy maintenance [24]. BMI is widely recognized as a key indicator of obesity in the medical field, particularly in studies related to reproductive health and pregnancy outcomes. After TA, BMI is considered a crucial factor influencing pregnancy prognosis. Research indicates that patients with a BMI ≤ 25 have a significantly higher pregnancy rate after reversal surgery (85.4%) compared to those with a BMI > 25 (65.9%) [25]. Elevated BMI may affect postoperative pregnancy outcomes through multiple mechanisms, primarily obesity-related metabolic disturbances such as insulin resistance, hormonal imbalances, and lipid metabolism abnormalities, all of which can negatively impact ovarian function, embryo development, and pregnancy maintenance [26]. Additionally, obesity, especially excessive visceral adipose tissue (VAT), has been strongly linked to reproductive hormone imbalances, increasing the risk of ovulatory dysfunction and implantation failure [27]. Moreover, obesity is commonly observed in patients with PCOS, and an elevated BMI can aggravate PCOS-associated metabolic dysfunctions, further heightening the risk of infertility [28].

In addition to advanced age and BMI, other factors related to tubal anastomosis may also influence pregnancy outcomes. For example, the duration of sterilization reflects the extent of tubal damage and its impact on fertility. Studies indicate that longer sterilization periods are associated with poorer pregnancy outcomes after surgery [29]. Furthermore, reproductive tract polyps [30], endometriosis [31], the extent of intraoperative or postoperative pelvic adhesions, postoperative recovery status, a history of abdominal surgeries, and tubal inflammation [32] may all affect pregnancy prognosis. However, current research on these factors remains limited. Future investigations involving large-scale, multicenter cohorts are needed to further clarify their specific impact on pregnancy outcomes following LTA.

The novelty of this study lies in three key aspects: it provides a comprehensive evaluation of risk factors for pregnancy loss after LTA, an area that has been explored in prior studies but not always using multivariate modeling; it employs a RCS model to reveal the pattern of influencing factors; and it validates the clinical utility of the predictive model through DCA. Nevertheless, several limitations must be acknowledged. Firstly, the single-center design of this study restricts the generalizability of the findings to broader populations, as results derived from a single center may not fully capture the heterogeneity of patient characteristics and clinical practices across diverse regions or healthcare environments. This limitation should be carefully considered when evaluating the applicability of our findings. Furthermore, although 640 patients were initially enrolled in this study, only 253 were ultimately analyzed, which introduces the potential for selection bias. Additionally, the absence of detailed surgical parameters limits the depth of mechanistic exploration, while the lack of long-term follow-up data constrains the evaluation of sustained outcomes. Finally, the relatively small sample size may diminish the reliability of subgroup analyses. It is also important to note that the exclusion criteria used in this study may impact the generalizability of the results. We excluded patients with contraindications to pregnancy (such as severe cardiovascular disease, hypertension, or severe renal disease), those with abnormal tubal anatomy discovered during surgery, male partners with infertility factors. And these factors are associated with lower rates of live births. Thus, excluding these patients may limit the reflection of these critical risk factors in our sample, and this limitation should be carefully considered when evaluating the broader applicability of our findings.

From a clinical perspective, this study provides valuable insights for postoperative management after LTA. First, it underscores the importance of comprehensive preoperative assessment for advanced-age and obese patients. Second, it highlights the need for enhanced pregnancy monitoring, particularly in high-risk populations. Finally, the findings offer data-driven support for individualized counseling by clinicians. Future research should focus on standardized evaluation of surgical techniques, mechanistic investigations at the molecular level, and multicenter prospective studies to further optimize pregnancy outcomes in LTA patients.

## 5. Conclusion

This study demonstrated that advanced maternal age and elevated BMI are significant risk factors for pregnancy loss following LTA, exhibiting distinct linear and nonlinear associations, respectively. These findings underscore the importance of personalized preoperative counseling and vigilant postoperative monitoring, especially for older and obese patients.

## Data Availability

The datasets generated are available from the corresponding author on reason-able request.
